# The endoplasmic reticulum-localized Ca^2+^-ATPase *OsACA5* regulates immunity and the seed setting rate in rice

**DOI:** 10.3389/fpls.2026.1758629

**Published:** 2026-02-05

**Authors:** Min Zhang, Yong Zhang, Zhirong Peng, Shanjun Tang, Zaireng Zhang, Chenming Liu, Xiao Luo, Junjie Xing

**Affiliations:** 1 Longping Agricultural College, Hunan University, Changsha, China; 2 State Key Laboratory of Hybrid Rice, Hunan Hybrid Rice Research Center, Hunan Academy of Agricultural Sciences, Changsha, China; 3 Hunan Institute of Nuclear Agriculture Sciences and Chinese Herbal Medicines, Changsha, China; 4 Liuyang Agriculture and Rural Bureau, Liuyang, China; 5 School of Food and Liquor, Sichuan Province Engineering Technology Research Center of Liquor-Making Grains, Sichuan University of Science and Engineering, Yibin, China

**Keywords:** Ca^2+^-ATPase, calcium signaling, rice, rice blast, seed setting rate

## Abstract

Calcium signaling plays a central role in plant immunity and development, and its homeostasis relies on the precise regulation of calcium transporters such as Ca^2+^-ATPases. However, the mechanisms by which Ca^2+^-ATPases coordinate disease resistance and reproductive development in rice remain largely unclear. In this study, we investigated the function of the endoplasmic reticulum (ER)-localized Ca^2+^-ATPase gene Oryza sativa autoinhibited Ca^2+^-ATPase 5 (OsACA5). The expression of OsACA5 was induced by infection with the rice blast fungus Magnaporthe oryzae and by the pathogen-associated molecular pattern (PAMP) flg22. In contrast, loss-of-function osaca5 mutants exhibited significantly enhanced resistance to rice blast, as evidenced by reduced lesion areas, increased reactive oxygen species (ROS) production, and elevated expression of defense-related genes, indicating that OsACA5 acts as a negative regulator of plant immunity. Further analyses revealed that OsACA5 negatively regulates early PAMP-triggered immunity (PTI)-associated Ca^2+^ influx induced by flg22 and chitin, thereby suppressing immune activation. In addition to enhanced disease resistance, osaca5 mutants displayed notable agronomic changes, including reduced seed setting rate and plant height, as well as increased thousand-grain weight and grain length. Together, these findings demonstrate that OsACA5 plays a critical role in balancing disease resistance and reproductive development in rice by modulating PTI-associated calcium signaling, providing new insights into the regulatory function of ER-localized Ca^2+^-ATPases and offering a potential strategy for breeding rice varieties with stable disease resistance and optimized yield-related traits.

## 1 Introduction

Calcium signaling is involved in physiological processes during plant growth, and affects cell growth, development, and stress responses and triggers systemic defense reactions (Tian et al., 2020). Ca^2+^ influx is among the earliest signaling events in response to stimulation by pathogen/microbe-associated molecular patterns (Grant et al., 2000). Ca^2+^ is a key second messenger in plant immunity, and increases in the cytosolic Ca^2+^ concentration occur in response to both PTI and effector-triggered immunity (ETI). These Ca^2+^ signals are subsequently decoded by Ca^2+^-binding sensor proteins, regulating multiple cellular processes involved in plant immunity (Koster et al., 2022). Ca^2+^-dependent phosphorylation of respiratory burst oxidase homologs (RBOHs) leads to ROS bursts, increasing plant resistance to biotic stress (Gilroy et al., 2016; Wang et al., 2019). *ROD1*(resistance of rice to diseases 1), a Ca^2+^ sensor, suppresses rice immunity by activating catalase to scavenge ROS; disruption of *ROD1* results in broad-spectrum disease resistance in rice Gao et al., 2021). Cytosolic Ca^2+^ bursts not only activate ROS but also regulate the expression of related disease resistance genes and MAPK kinase activity (Kim et al., 2009; Ranf et al., 2011). Under specific conditions, leaf infiltration with Ca^2+^ ionophores can induce high levels of PR gene expression in *cpn1-1*(COPINE1) plants (Lee and McNellis, 2009). Recent transcriptome-based analyses of rice responses to *Magnaporthe oryzae* infection further highlight Ca^2+^-related signaling genes as central immune hubs, emphasizing the importance of Ca^2+^ dynamics in rice blast interactions (Salem et al., 2025a).

In plants, calcium ions are transported primarily by three types of proteins: channels, pumps, and exchangers. In addition, several nonspecific calcium transport proteins, including cyclic nucleotide-gated channels (CNGCs), glutamate receptor-like proteins (GLRs), and annexins (ANNs), have been identified (Goel et al., 2011). Ca^2+^-ATPases are divided into two subfamilies: P-type IIA and P-type IIB. *Oryza sativa* ER-type Ca^2+^-ATPases (*OsECAs*), which belong to the P-type IIA subfamily, are primarily localized to the plasma membrane. They transport ions such as calcium, manganese, and cadmium and are associated with stress responses (Li et al., 2008). The *Os*ACA family includes type IIB P-type autoinhibited Ca^2+^-ATPases, which have been identified in multiple species Axelsen and Palmgren, 2001; Taneja and Upadhyay, 2018; Jiang et al., 2023). Previous studies have identified twelve *At*ACA genes Chandan et al., 2024). *At*ACA family genes have diverse functions. The first gene identified, *AtACA1* was confirmed to be localized to the chloroplast inner envelope membrane and plays a role in controlling the stomatal aperture and signal transduction between chloroplasts and the cytoplasm (Harper et al., 1998; Rahmati Ishka et al., 2021). *AtACA7*, *AtACA9*, and *AtACA13* regulate fertility in *Arabidopsis* by participating in pollen and pollen tube development (Schiott et al., 2004; Yu et al., 2018; Rahmati Ishka et al., 2021). *At*ACAs can also regulate stress resistance in *Arabidopsis*; furthermore, *AtACA12* expression is significantly increased after flg22 treatment (Garcia Bossi et al., 2020). The expression levels of *AtACA4*, *AtACA8*, and *AtACA10* change significantly under abiotic stresses such as salt and cold (Yang et al., 2017; Yu et al., 2018). The simultaneous loss of *Arabidopsis AtACA4* and *AtACA11* leads to decreased disease resistance Boursiac et al., 2010), suggesting that *Os*ACAs might regulate rice immunity through a similar mechanism; however, this hypothesis awaits experimental verification.

Research on the *Os*ACA family in rice has focused primarily on resistance to abiotic stress. *OsACA1* and *OsACA8* increase drought resistance by regulating lignin synthesis and stomatal closure in rice (Kan et al., 2017; Bang et al., 2019). *OsACA6* is regulated by miR1432 to modulate resistance to cold, salt, and drought stress in rice Dai et al., 2024). Moreover, the expression of *OsACA6* and *OsACA7* increases under acid rain stress (Liang and Ma, 2025). While genes in the *Os*ACA family play regulatory roles in various aspects of abiotic stress, reports on their role in biotic stress are limited, as only *OsACA9* has been reported to regulate resistance to bacterial blight through the accumulation of ROS and to positively regulate leaf senescence in rice (Wang et al., 2024). *Os*ACA family proteins primarily regulate plant growth by modulating cytosolic and extracellular Ca^2+^ concentrations, but their functions in disease resistance remain to be elucidated.

Although the role of calcium signaling in plant immunity has been extensively studied, the specific function of ER-localized calcium ATPases, such as *OsACA5*, in immune responses remains unclear. Integrative multitranscriptomic analyses of *rice–M. oryzae* interactions have also revealed endoplasmic reticulum (ER)-associated processes as important components of defense responses, suggesting that a closer examination of ER-localized Ca^2+^ transport machinery during infection is warranted (Salem et al., 2025b). Previous research has focused primarily on the role of calcium ATPases in stress responses, whereas the dual function of *OsACA5* in both immunity and reproductive development has not been thoroughly explored.

This study addresses this gap by revealing the negative regulatory role of *OsACA5* in immune responses, in addition to its positive regulation of reproductive development. We found that *OsACA5* is localized to the ER and that *osaca5* mutants exhibit enhanced resistance to blast disease, which is accompanied by increases in calcium influx, ROS accumulation, and the expression of defense-related genes. These mutants presented significantly reductions in seed setting rate and plant height, among other changes in agronomic traits. By elucidating how *OsACA5* influences both immunity and reproduction through the regulation of calcium homeostasis, this research provides new insights for crop breeding aimed at increasing disease resistance and improving seed setting rates.

## 2 Results

### 2.1 *OsACA5* expression is induced by *M. oryzae* and flg22 treatment

**Figure 1:**
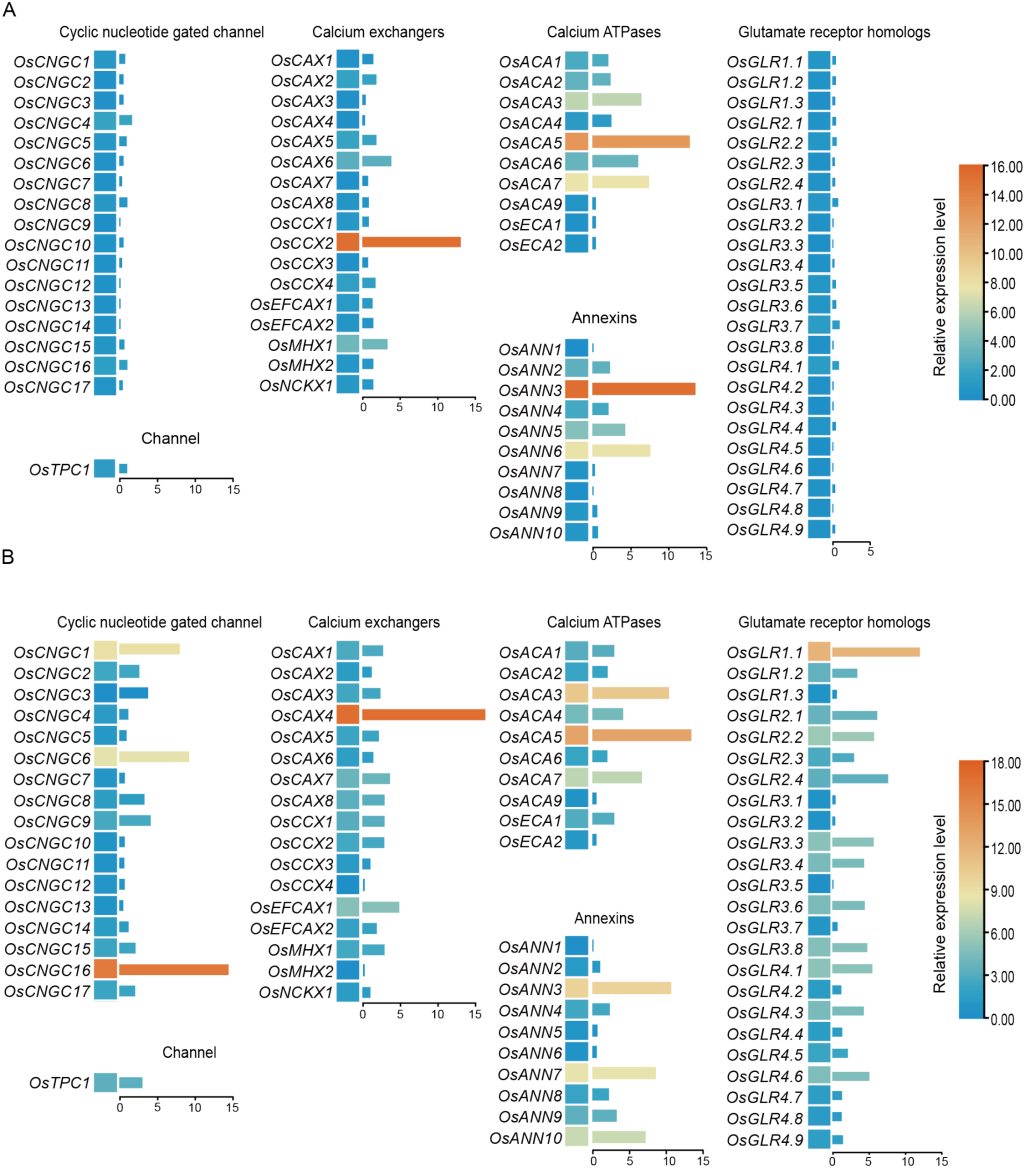
The expression of *OsACA5* is induced by *Magnaporthe oryzae* and the pathogen-associated molecular pattern flg22. **(A)** Heatmap and corresponding bar plots showing the relative expression levels of selected calcium transporter genes in NIP plants at 24 h postinoculation with *M. oryzae* strain 70–15 compared with NIP plants at 0h **(B)** Heatmap and corresponding bar plots showing the relative expression levels of the same genes in NIP plants at 1 h after treatment with 1 μM flg22 compared with those in the water control. Gene expression levels were determined by RT–qPCR and are presented as relative expression values. Bar plots show the mean ± SD of three independent biological replicates (n = 3). The heatmaps and adjacent bar plots represent the same datasets. Red and blue colors indicate upregulation and downregulation, respectively, as shown in the color scale.

The presence of calcium transport elements in the rice genome has been reported previously (Singh et al., 2014). To identify calcium signaling components involved in the rice immune response, we analyzed the expression patterns of different calcium transporter gene families, including cation/H^+^ exchangers (CAXs), autoinhibited Ca^2+^-ATPases (ACAs), annexins (ANNs), glutamate receptor-like channels (GLRs), and cyclic nucleotide-gated channels, in rice following *M. oryzae* infection and flg22 treatment. Upon infection with *M. oryzae*, the expression of genes belonging to the GLR and CNGC families was not induced, whereas several genes in the other three families were upregulated to varying degrees. Among them, the expression of *OsCCX2*, *OsACA5*, and *OsANN3* was most strongly induced. Under flg22 treatment, several members of all five gene families were upregulated to varying extents, with *OsCNGC16*, *OsCAX4*, *OsACA5*, *OsGLR1.1*, and *OsANN3* exhibiting the greatest degree of induction (**Figures 1A, B**). Taken together, these results indicate that several calcium-related genes respond to both *M. oryzae* infection and flg22 treatment. Although *OsANN3* (LOC_Os05g31750) was also induced under both conditions, this gene has been previously characterized and shown to regulate ROS production and Ca^2+^ influx dynamics, and was therefore not further analyzed in this study (Zhang et al., 2021). Within the OsACA family, the expression of *OsACA3*, *OsACA5*, and *OsACA7* was induced by both treatments. Among these genes, OsACA5 exhibited the highest expression level, whereas *OsACA3* showed the second-highest expression. On the basis of their strong and consistent transcriptional responses, *OsACA5* and *OsACA3* were selected for further functional analysis.

### 2.2 *OsACA5* is localized to the ER

**Figure 2:**
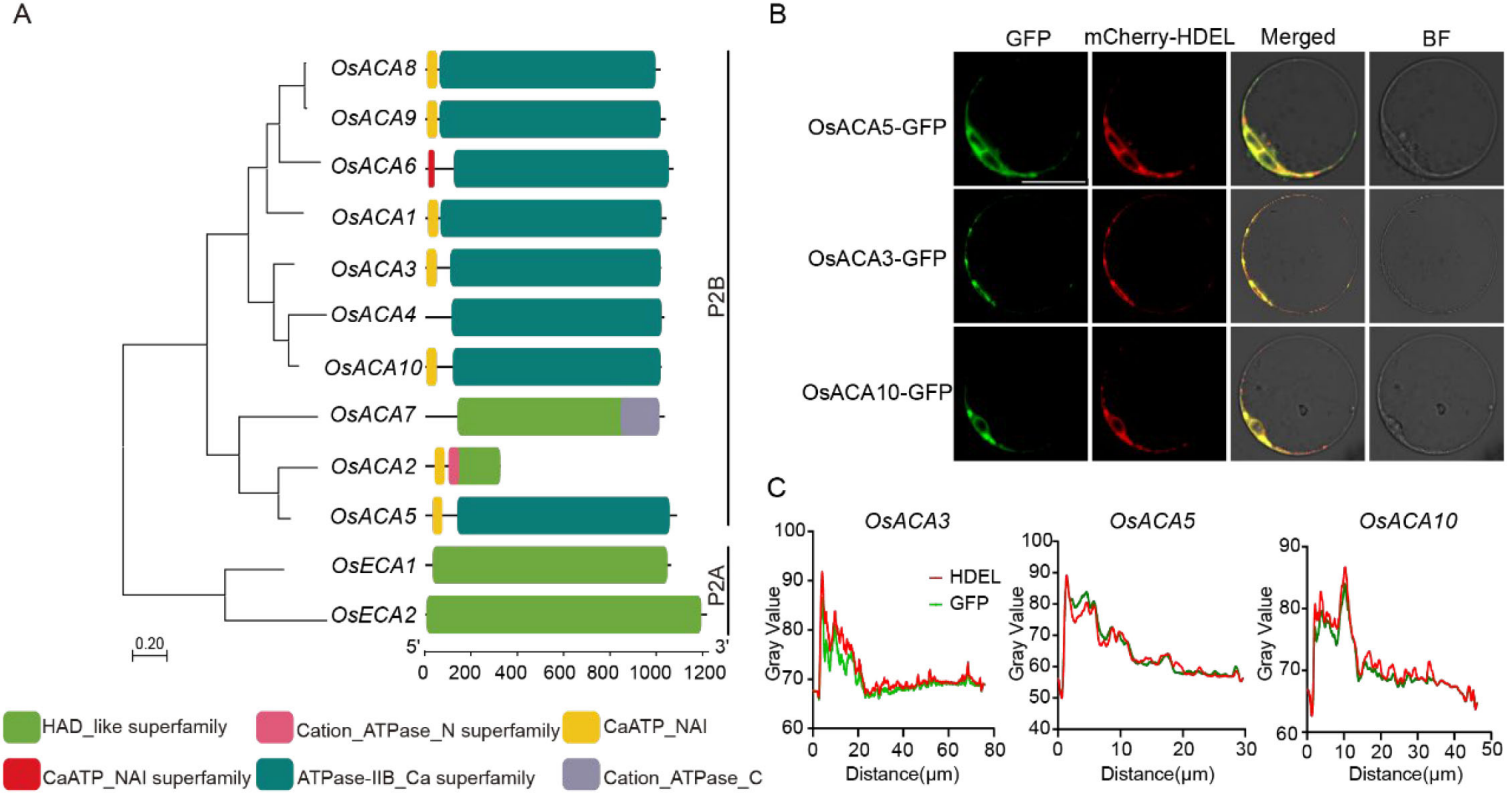
Subcellular localization of rice OsACA family members in protoplasts. **(A)** Phylogenetic relationship and domain architecture of the rice OsACA family. The phylogenetic tree is shown on the left, and the corresponding domain organization of each protein is shown on the right. Colored boxes indicate conserved domains. **(B)** Confocal images of OsACA3–GFP, OsACA5–GFP and OsACA10–GFP (green) co-expressed with the endoplasmic reticulum marker mCherry–HDEL (red) in rice protoplasts. The GFP channel, mCherry–HDEL channel, merged images (Merged), and bright-field images (BF) are shown. Scale bar = 10 μm. **(C)** Fluorescence intensity profiles of GFP (green) and mCherry–HDEL (red) were extracted using the entire image area as the region of interest (ROI). Gray values are plotted against distance (μm) to assess the degree of spatial overlap between the two signals.

To investigate the potential functions of *OsACA3* and *OsACA5*, we performed a comprehensive analysis of the rice Ca^2+^-ATPase gene family, including an analysis of phylogenetic relationships and domain architecture, *cis*-acting element prediction, and spatiotemporal expression profiling (**Figures 2A**; **Supplementary Figures S2, 3**). While promoter *cis*-acting element analysis, spatiotemporal expression profiling, and conserved motif distribution analysis revealed considerable diversity among family members, domain architecture analysis revealed that *OsACA3*, *OsACA5*, and *OsACA10* share an identical domain structure. This specific domain organization differs notably from that of *OsACA6* and *OsACA7*; previous studies reported that *OsACA6* is localized to the plasma membrane, whereas *OsACA7* is localized to the Golgi apparatus (Huda et al., 2013; Singh et al., 2014). To further validate the subcellular localization of *OsACA6*, we co-expressed OsACA6–GFP with the ER marker mCherry–HDEL in rice protoplasts and performed a co-localization analysis (**Supplementary Figure S5**). OsACA6–GFP fluorescence was mainly detected at the cell periphery, whereas mCherry–HDEL showed a typical reticulate ER network pattern. Consistent with these distinct distribution patterns, only limited overlap between OsACA6–GFP and mCherry–HDEL signals was observed, supporting that *OsACA6* is unlikely to be ER-localized. To verify the subcellular localization of the candidate genes, we constructed OsACA3–GFP and *Os*ACA5–GFP fusion protein and transiently expressed them in rice protoplasts. The dataconfirmed that *OsACA3* and *OsACA5* are localized to the ER, which is consistent with the localization of the ER marker protein HDEL (Napier et al., 1992). Additional experiments revealed that the structurally similar gene *OsACA10* is also localized to the ER (**Figure 2B**). Moreover, fluorescence intensity line-scan analysis along the indicated regions showed highly overlapping profiles between the GFP signal and the HDEL marker signal, with coincident peaks across the scanned distances (**Figure 2C**), further supporting ER localization of *OsACA3*, *OsACA5*, and *OsACA10*. Together, these results support the ER localization of *OsACA3*, *OsACA5*, and *OsACA10*. Given that *OsACA10* shares an identical domain architecture with *OsACA3* and *OsACA5* (**Figure 2A**), we propose that this specific domain organization featuring the CaATP_NAI and ATPase_IIB_Ca domains may be associated with ER targeting in the OsACA family. Future studies are needed to test whether other members possessing this architecture (*OsACA1*, *OsACA8*, and *OsACA9*) are also ER-localized.

### 2.3 *OsACA5* negatively regulates rice blast resistance

**Figure 3:**
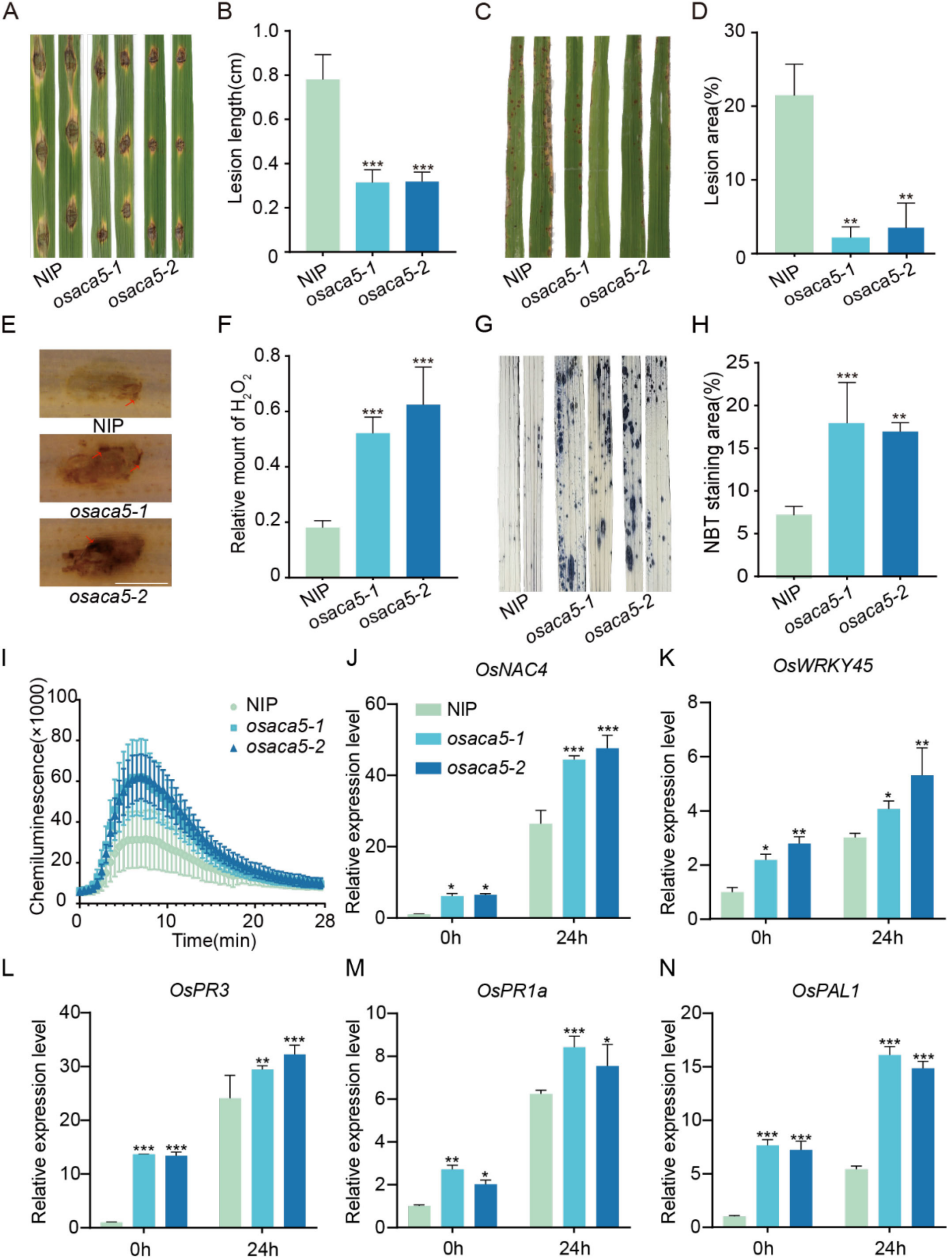
The *osaca5* mutant exhibits increased resistance to *M. oryzae* and potentiated immune responses. **(A)** Phenotypes of NIP and *osaca5* plants at 7 days after inoculation (by wounding) with the compatible *M. oryzae* isolate 70-15 (n = 15 lesions). **(B)** Quantitative analysis of lesion areas from the wound inoculation experiment shown in **(A)**. **(C)** Phenotypes of NIP and *osaca5* plants at 7 days after spray inoculation with the compatible *M. oryzae* isolate 70-15 (n = 15 lesions). **(D)** Quantitative analysis of diseased leaf area from the spray inoculation experiment shown in **(C)**. **(E)** Detection of hydrogen peroxide (H_2_O_2_) accumulation by 3,3’-diaminobenzidine (DAB) staining in the leaves of NIP and *osaca5* plants at 48 hours post-inoculation (hpi) with *M. oryzae*; reddish-brown staining indicates H_2_O_2_. **(F)** Quantification of the DAB-stained area in **(E)**. **(G)** Detection of superoxide anion accumulation by nitroblue tetrazolium (NBT) staining in the leaves of NIP and *osaca5* plants at 48 hpi with *M. oryzae*; blue staining indicates superoxide. **(H)** Quantification of the NBT-stained area in **(G)**. **(I)** Kinetics of reactive oxygen species (ROS) bursts, determined by measuring luminescence, in the leaf discs of NIP and *osaca5* plants after treatment with 1 μM flg22. **(J–N)** Relative expression levels of the defense-related genes *OsNAC4***(J)**, *OsWRKY45***(K)**, *OsPR3***(L)**, *OsPR1a***(M)**, and *OsPAL1***(N)** in NIP and *osaca5* plants at 0 and 24 hours post-inoculation with *M. oryzae*, as determined by qRT–PCR. The data in **(B, D, F, H, I)** are presented as the mean ± SD (n ≥ 3). Asterisks above the bars (or at the peak time points in I) indicate statistically significant differences according to one-way ANOVA followed by Dunnett’s multiple comparisons test (*p < 0.05, **p < 0.01, ***p < 0.001, ns>0.05). Scale bar in **(E)** = 200 µm.

To explore the potential roles of *OsACA3* and *OsACA5* in rice immunity, we generated mutants on the NIP background, obtaining two independent mutant lines for each gene, namely, *osaca5-1*, *osaca5-2*, *osaca3–1* and *osaca3-2* (**Supplementary Figure S1**). Lesion areas were investigated seven days after wound inoculation with *M. oryzae* strain 70-15. The lesion area in NIP plants was significantly larger (by 40%) than that in *osaca5* plants indicating that *OsACA5* deficiency increases resistance to blast. Supsequent spray inoculation confirmed these results, with the diseased area in *osaca5* plants reaching only nearly 15% of that in NIP plants (**Figures 3A–D**). Both the wound and spray inoculation experimental results revealed that *osaca5* increases rice blast resistance.

In contrast, wound-inoculation assays of two independent *osaca3* mutant lines (*osaca3–1* and *osaca3-2*) revealed lesion areas that were not significantly different from those of NIP plants (**Supplementary Figures S4A, B**), indicating that *OsACA3* deficiency does not alter rice blast resistance under these conditions. After establishing the resistant phenotype of *osaca5*, we assessed the transcript levels of other OsACA family members in *osaca5*.Compared with that of NIP, although the expression of most *OsACA* genes did not significantly change, and only minor decreases were observed for a few members, the expression of *OsACA5* markedly decreased (**Supplementary Figure S4C–K**).

To clarify the role of *OsACA5* in rice blast resistance, immune physiological indicators were detected in NIP and *osaca5* plants after inoculation with 70-15. DAB and NBT staining at 48 hours post-inoculation revealed significantly larger areas stained dark brown and blue–purple in *osaca5* plants than in NIP plants (**Figures 3E–H**). We also used chemiluminescence to assess the ROS burst in *osaca5* plants after stimulation. Upon treatment with flg22, compared with NIP plants, *osaca5–1* and *osaca5–2* plants presented stronger ROS bursts (**Figure 3I**). We subsequently examined the expression levels of defense-related genes to further investigate the immune response and found that a series of defense-related genes were activated; the expression levels of *Oryza sativa* NAC domain-containing transcription factor 4 (*OsNAC4*), *Oryza sativa* WRKY transcription factor 45 (*OsWRKY45*), *Oryza sativa* pathogenesis-related protein 3 (*OsPR3*), *Oryza sativa* pathogenesis-related protein 1a (*OsPR1a*), and *Oryza sativa* phenylalanine ammonia-lyase 1 (*OsPAL1*) were significantly greater in *osaca5* plants than in NIP plants at both 0 hours and 24 hours post-inoculation (**Figures 3J–N**). The increase in ROS activity, oxidative bursts, and defense-related genes expression in *osaca5* plants upon pathogen inoculation indicate that *OsACA5* negatively regulates the immune response in rice.

### 2.4 *OsACA5* negatively regulates PAMP-triggered calcium influx and affects the expression of calcium signaling-related genes

**Figure 4:**
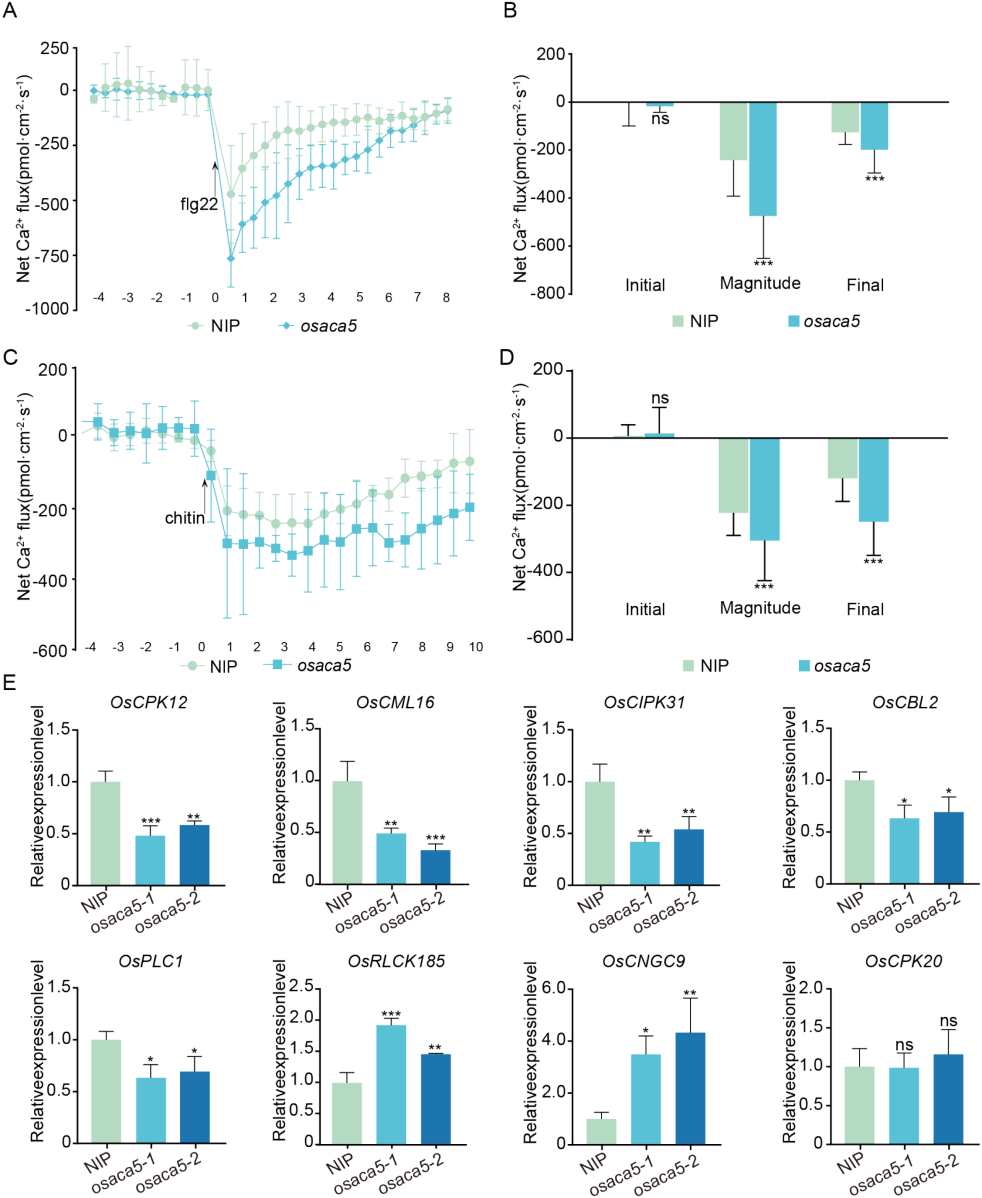
*OsACA5* negatively regulates calcium signaling. **(A)** Net Ca^2+^ flux in rice mesophyll cells measured by noninvasive microtest technology (NMT) after treatment with 1 μM flg22. The arrow indicates the time of flg22 application. **(B)** Statistical analysis of the Ca^2+^ flux rates at the initial, peak (magnitude), and final stages from the experiment shown in **(A)**. **(C)** Net Ca^2+^ fluxes in rice mesophyll cells after treatment with 1 μM chitin. The arrow indicates the time of chitin application. **(D)** Statistical analysis of the Ca^2+^ flux rates at the initial, peak (magnitude), and final stages from the experiment shown in **(C)**. **(E)** Relative expression levels of calcium signaling-related genes (*OsCPK12, OsCML16, OsCIPK31, OsCBL2, OsPLC1, OsRLCK185, OsCNGC9*, and *OsCPK20*) in NIP and *osaca5* plants, as determined by qRT–PCR. Asterisks indicate statistically significant differences between NIP and *osaca5* plants at the indicated time points (Student’s t test; *p < 0.05, **p < 0.01, ***p < 0.001). The data in **(E)** are presented as the mean ± SD (n = 3). Different lowercase letters above the bars indicate statistically significant differences according to one-way ANOVA followed by Dunnett’s multiple comparisons test (*p* < 0.05).

To investigate whether *OsACA5* mediates Ca^2+^ influx under PAMP stimulation, noninvasive microtest technology (NMT) was used to detect dynamic changes in Ca^2+^ in mesophyll cells after treatment with PAMPs (flg22 and chitin), which have been shown to trigger PTI signaling in plants Chinchilla et al., 2007). Under untreated conditions, both NIP and *osaca5* plants showed small Ca^2+^ fluxes, maintaining a stable state of Ca^2+^ influx/efflux. After the addition of 1 µM flg22, Ca^2+^ influx increased sharply. Compared with that in NIP plants, the Ca^2+^ flux in *osaca5* plants was significantly greater, peaking rapidly within a short time and slowly returning to the initial state after 8 minutes (**Figures 4A, B**). After the addition of 1 µM chitin, Ca^2+^ influx increased sharply in both NIP and *osaca5* plants, with the Ca^2+^ influx in *osaca5* plants being significantly greater than that in NIP plants, although the intensity of Ca^2+^ influx was less than that after flg22 treatment (**Figures 4C, D**). Both flg22 and chitin caused Ca^2+^ influx, and the degree of influx was significantly greater in *osaca5* plants than in NIP plants. Calcium influx is an early signal stimulated by PAMPs (Upadhyay, 2022). The stronger and more sustained Ca^2+^ influx observed in *osaca5* plants may contribute to the heightened activation of immune responses and ultimately to the enhanced resistance of *osaca5* plants to *M. oryzae*.

To validate the above results, we selected several calcium transporter genes for qRT–PCR analysis. We compared the expression levels of genes encoding calcium-dependent protein kinases (*Oryza sativa* calcium-dependent protein kinase 12 (*OsCPK12*) (Tavu and Redillas, 2025) and *OsCPK20*Fu et al., 2013)), calcium signaling sensors (*Oryza sativa* calmodulin-like protein 16 (*OsCML16*) (Liu et al., 2025) and *Oryza sativa* calcineurin B-like protein 2 (*OsCBL2*) (Qin et al., 2024)), and calcium channel proteins (*OsCNGC9*) (Wang et al., 2021), and calcium signaling-related genes (*Oryza sativa* phosphoinositide-specific phospholipase C1 (*OsPLC1*) (Li et al., 2017), *Oryza sativa* CBL-interacting protein kinase 31 (*OsCIPK31*) (Piao et al., 2010), and *Oryza sativa* receptor-like cytoplasmic kinase 185 (*OsRLCK185*) (Yamaguchi et al., 2013)) between NIP and *osaca5* plants. Compared with those in NIP plants, the expression levels of *OsCPK12*, *OsCML16*, *OsCIPK31*, *OsCBL2*, and *OsPLC1* significantly decreased in *osaca5* plants, whereas the expression levels of *OsRLCK185* and *OsCNGC9* significantly increased. *OsCPK20* expression did not significantly differ (**Figure 4E**), indicating that *OsACA5* may indirectly influence the activity of other calcium channels.

### 2.5 *OsACA5* regulates agronomic traits in rice

**Figure 5:**
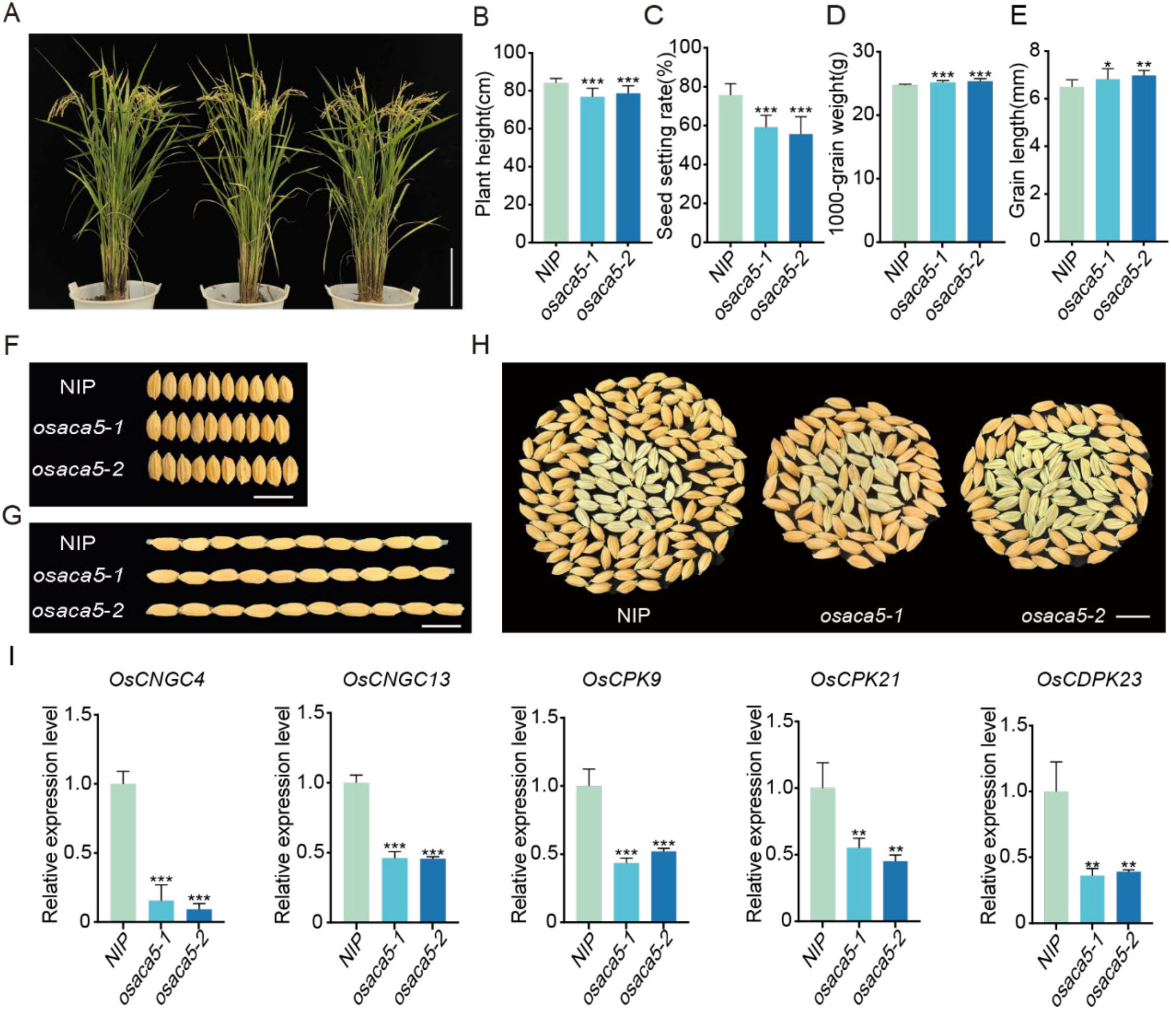
*OsACA5* is required for maintaining normal agronomic traits in rice. **(A)** Architecture of NIP and two independent *osaca5* mutant lines (scale bar = 10 cm). **(B)** Plant height of NIP and *osaca5* plants. **(C)** Seed setting rate of NIP and *osaca5* plants. **(D)** Thousand-grain weight of NIP and *osaca5* plants. **(E)** Grain length of NIP and *osaca5* plants. **(F)** Grain width phenotype of NIP and *osaca5* seeds (scale bar = 1 cm). **(G)** Grain length phenotype of NIP and *osaca5* seeds (scale bar = 1 cm). **(H)** Single panicle Grains arranged in circular patterns showing the numbers of filled and empty grains (scale bar = 1 cm). **(I)** Relative expression levels of Ca^2+^ signaling- and fertility-related genes in NIP and *osaca5* plants determined by qRT–PCR.Data in **(B–E, I)** are presented as mean ± SD (n ≥ 15 plants for B-E; n = 3 biological replicates for I). Statistical significance was determined as described in the Methods. *P < 0.05, **P < 0.01, and ***P < 0.001.

An increase in disease resistance in plants is often accompanied by a reduction in yield (Kim et al., 2024b). Therefore, we investigated the agronomic traits of the *osaca5* mutants. The height of the NIP plants was 84 cm, whereas those of the *osaca5–1* and *osaca5–2* plants were 76 cm and 78 cm, respectively. The height of the NIP plants was significantly greater than that of the *osaca5* plants (**Figures 5A, B**). The seed setting rate of the *osaca5* plants, below 60%, was significantly lower than that of the NIP plants (**Figure 5C**). The effective tiller number did not significantly differ between NIP and *osaca5* plants (**Supplementary Figure S6A**). With respect to grain length, compared with NIP plants, *osaca5* plants had significantly longer grains (**Figures 5E, G**). However, no significant difference in grain width was detected between the *osaca5* and NIP plants (**Figured 5F**, **S6B**). Additionally, the thousand-grain weight of the *osaca5* plants was significantly greater than that of the NIP plants (**Figure 5D**). A comparison of the total number of grains per panicle revealed that NIP plants produced far more grains than did *osaca5* plants, with *osaca5* plants did producing a greater proportion of empty grains (**Figure 5H**). These findings suggest that *osaca5* mutation severely affects the seed setting rate in rice. However, no significant differences in pollen viability or other traits were detected (**Supplementary Figures S6C, D**).

To determine whether disrupted Ca^2+^ signaling contributes to the reduced seed setting rate of *osaca5* plants, we examined the expression of several Ca^2+^ signaling- and fertility-related genes by qRT–PCR. These genes included the cyclic nucleotide-gated channel family members *OsCNGC4* and *OsCNGC13*, as well as the Ca^2+^-dependent protein kinase family members *OsCPK9*, *OsCPK21*, and *OsCDPK23*. Previous studies have shown that *OsCNGC*4 and *OsCNGC13* influence panicle fertility and the seed setting rate by regulating pollen germination and pollen tube growth within the pistil (Xu et al., 2017; Kim et al., 2024a), whereas *OsCPK9*, *OsCPK21*, and *OsCDPK23* act as typical Ca^2+^ signal “decoders” that finely modulate reproductive processes such as pollen development, panicle development, and grain formation Asano et al., 2002; Wei et al., 2014; Wen et al., 2019). Compared with those in NIP plants, the transcript levels of *OsCNGC4*, *OsCNGC13*, *OsCPK9*, *OsCPK21*, and *OsCDPK23* were significantly lower in both *osaca5–1* and *osaca5-2*, with the two allelic mutants displaying a highly consistent downward trend (**Figure 5I**). These results indicate that Ca^2+^ signaling pathways that are closely associated with reproductive development are globally suppressed in the absence of *OsACA5*, which may be an important cause of the markedly reduced seed setting rate observed in the *osaca5* mutants.

## 3 Discussion

Calcium signaling plays a crucial role in plant immune responses and is tightly regulated by calcium transporters, including autoinhibited Ca^2+^-ATPases (Tian et al., 2020). In this study, *OsACA5* expression was strongly induced by *M. oryzae* infection and flg22 treatment (**Figure 1**), suggesting that *OsACA5* may function in the early phase of PTI activation. Domain analysis of *Os*ACA family members revealed that *OsACA5* shares conserved CaATP_NAI and ATPase-IIB_Ca superfamily domains with *OsACA1*, *OsACA3*, *OsACA8*, *OsACA9*, and *OsACA10*. In contrast, *OsACA6* lacks the CaATP_NAI domain but contains a CaATP_NAI superfamily domain, whereas *OsACA7* has two distinct domains, the HAD_like superfamily and Cation_ATPase_C domains (**Figure 2A**). To assess whether domain composition is correlated with subcellular localization, we examined the expression of both *OsACA3* and *OsACA10*,which have the same domains as *OsACA5* does. Our analysis revealed that both *OsACA3* and *OsACA10* localized to the ER (**Figure 2B**). In contrast, *OsACA6* and *OsACA7*, which have distinct domain structures, were localized to the plasma membrane (**Supplementary Figure S5**) and Golgi apparatus, respectively. On the basis of these results, we hypothesize that other members of the OsACA family, such as *OsACA1*, *OsACA8*, and *OsACA9*, may also localize to the ER and that the conserved CaATP_NAI domain could play a role in determining ER localization. However, further studies are needed to verify this hypothesis. The ER is a crucial site for protein synthesis, folding, and calcium storage. *OsACA5* plays a critical role in maintaining ER calcium homeostasis, which is essential for both immune signaling and reproductive development. *osaca5* mutation may disrupt ER calcium homeostasis, potentially triggering the unfolded protein response (UPR) and thereby amplifying plant immune signaling (Senft and Ronai, 2015). Although *osaca5* mutation altered calcium flux (**Figures 4A–D**), the precise mechanism through which ER-localized calcium ATPases modulate calcium signaling through membrane-associated effector components remains unclear.

In *osaca5* plants, the expression of *OsCPK12*, *OsCML16*, *OsCIPK31*, *OsCBL2*, and *OsPLC1* was downregulated, whereas that of *OsRLCK185* and *OsCNGC9* was upregulated, suggesting that the Ca^2+^ signaling network was disrupted (**Figure 4E**). The decreased expression of Ca^2+^ sensors and kinases may weaken calcium decoding, whereas the induction of *OsRLCK185* and *OsCNGC9* may represent a compensatory response to sustain Ca^2+^ influx and defense activation. Together, these findings imply that *OsACA5* acts as an ER-localized Ca^2+^ pump that connects ER calcium homeostasis with cytosolic Ca^2+^ signaling and immune regulation in rice.

To investigate the function of *OsACA5*, the lesion area of the *osaca5* plants inoculated with *M. oryzae* was investigated and found to be significantly smaller than that of the NIP plants (**Figures 3A–D**). *osaca5* mutation enhanced disease resistance. *osaca5* plants exhibited greater H_2_O_2_ accumulation after *M. oryzae* infection (**Figures 3E–H**) and exhibited stronger ROS bursts under flg22 treatment (**Figure 3I**), and the expression of a series of core defense-related genes was significantly greater in *osaca5* plants than in NIP plants (**Figures 3J–N**). Analysis of the phenotypes and physiological behaviors of both types of plants confirmed that *OsACA5* is a negative immune regulator. *OsACA5* likely negatively regulates rice blast resistance by modulating calcium signaling. An increase in the cytosolic Ca^2+^ concentration is closely related to ROS burst (Tan et al., 1998). Using NMT, we found that Ca^2+^ influx in *osaca5* plants was significantly greater than that in wild-type plants after flg22 and chitin treatment (**Figures 4A–D**). *osaca5* mutation may impair the active transport of Ca^2+^ into the ER lumen. Upon PAMP stimulation, massive Ca^2+^ influx leads to a substantial increase in the cytosolic Ca^2+^ concentration, subsequently triggering a downstream ROS burst and sustained elevation of the expression of defense genes.

However, the enhanced immunity in *osaca5* was accompanied by a decreased seed setting rate, reduced plant height, and fewer grains per panicle (**Figure 5**). *osaca5* mutation might directly cause these defects by disrupting specific growth and development processes, particularly reproductive development. Previous studies have shown that inhibiting the ER calcium pump in *Arabidopsis* leads to depletion of ER calcium stores and disruption of cytosolic calcium homeostasis and consequently blocks pollen tube growth (Iwano et al., 2009). Similarly, research on *Oryza sativa* has shown that RALF signaling establishes a calcium gradient by activating MLO calcium channels to maintain pollen tube integrity and directional growth (Gao et al., 2023). Through pollen iodine staining assays, we found that pollen viability was not impaired in *osaca5* plants (**Supplementary Figures S6C, D**). We therefore speculate that the reduced seed setting rate in the mutants may have been caused by altering processes such as pollen tube guidance or early postfertilization development rather than directly regulating pollen viability.

In this study, we found that compared with wild-type plants, *OsACA5* mutants exhibit enhanced immune responses, likely due to increased calcium influx and ROS bursts, but their seed setting rate is lower (**Figure 5**). This suggests that enhanced immunity may come at the cost of reproduction, reflecting a physiological trade-off where resources are allocated to immune responses during pathogen infection, which may suppress reproductive processes. We speculate that *OsACA5* deficiency disrupts ER calcium homeostasis, affecting pollen tube calcium balance and impairing pollen tube growth or embryo development, ultimately leading to reduced seed setting. These findings indicate that while *OsACA5* enhances immunity, its normal function is essential for reproductive development, and plants may need to balance immunity and reproduction under stress conditions. Future studies will further explore how *OsACA5* coordinates these processes and regulates calcium signaling.

Taken together, the results of this study reveal the significant role of the ER-localized Ca^2+^-ATPase *OsACA5* in regulating immunity and development in rice. *OsACA5* prevents the activation of immune responses by negatively regulating pathogen signal-triggered calcium influx and ROS bursts. Moreover, its normal function is vital for maintaining calcium homeostasis during reproductive processes, thereby ensuring yield formation. *osaca5* mutation results in increased disease resistance but a reduced seed setting rate. This study demonstrates that the ER-localized Ca^2+^-ATPase *OsACA5* modulates the regulation of disease resistance and the seed setting rate in rice. The *osaca5* mutants exhibited increases in calcium influx, and disease resistance, and, consequently, a reduced seed setting rate.

## 4 Materials and methods

### 4.1 Plant materials and growth conditions

Transgenic lines were developed on the Nipponbare (NIP) background and maintained in our laboratory. Two independent *osaca5* knockout mutants (*osaca5–*1 and *osaca5*-2) were generated using CRISPR/Cas9. Guide RNAs targeting *OsACA5* were designed using the E-CRISP web tool and cloned and inserted into a Cas9 expression vector. Two sgRNAs were used to target *OsACA5*, and the resulting mutants contained indels at least two target sites, as shown in **Supplementary Figure S1**. The constructs were introduced into NIP calli via *Agrobacterium tumefaciens* EHA105-mediated transformation, and T_0_ plants were screened by PCR and sequencing of the *OsACA5* target site. T_1_ and T_2_ plants of the two knockout mutants and wild-type NIP were grown under field conditions at the Yazhou Bay Certification and Breeding Base in Sanya, Hainan Province. At maturity, the agronomic traits of ten randomly selected plants from each genotype were assessed. After harvest, the grains were dried at 37 °C for four days, after which the seed setting rate and thousand-grain weight were measured.

### 4.2 Phylogenetic tree construction, domain analysis, cis-acting element prediction, and spatiotemporal expression profiling

To investigate the evolutionary relationships and functional characteristics of the rice Ca^2+^-ATPase gene families OsACA and OsECA, we performed gene family identification and phylogenetic analysis, domain analysis, cis-acting element prediction, and spatiotemporal expression profiling for both families. First, a phylogenetic tree was constructed using the full-length amino acid sequences of members from the OsACA and OsECA families. The tree was generated in MEGA using the Neighbor-Joining (NJ) method, and its stability was evaluated by 1000 bootstrap resamplings. The resulting phylogeny reveals the evolutionary relationships among members of the two families and provides a basis for further functional inference. For domain analysis, the amino acid sequences of OsACA and OsECA proteins were analyzed using InterPro, SMART, and NCBI CD-Search (Conserved Domain Database, CDD) to identify conserved domains, such as the P-type ATPase domain and the CaATP_NAI domain. The domain information was integrated with the phylogenetic tree to further characterize the potential functional features of different gene members. In addition, cis-acting element prediction was conducted on the 2.0 kb promoter regions upstream of each OsACA and OsECA gene using the PlantCARE database. The identified cis-acting elements were categorized and summarized, and the results were visualized using bar graphs. Finally, to investigate the spatiotemporal expression patterns of the OsACA and OsECA family members, we retrieved expression data from the RiceXPro database. A heatmap was generated based on transcript abundance (TPM) across different tissues and developmental stages, revealing the dynamic expression profiles of these Ca^2+^-ATPase genes throughout rice development.

### 4.3 Subcellular localization

The coding sequence of *OsACA5* lacking its stop codon was amplified and fused in frame to the N-terminus of GFP under the control of the CaMV 35S promoter in the pCAMBIA1390 vector. Recombinant and empty constructs were delivered into rice mesophyll protoplasts isolated from 10-day-old etiolated seedlings using a Plant Protoplast Preparation and Transformation Kit (Coolaber, Beijing, China; Cat. PPT111-5T) and transfected via a PEG-CaCl_2_ solution at room temperature. After overnight incubation at 28°C in the dark, GFP fluorescence was observed in live protoplasts on a Zeiss LSM880 confocal laser scanning microscope (Carl Zeiss AG, Oberkochen, Germany).

### 4.4 Pathogenicity assays

Seven- to ten-day-old cultures of *M. oryzae* strain 70–15 were washed from agar plates with 0.5% (v/v) Tween-20, and the conidial suspension was adjusted to 2.0 × 10^5^ spores/mL. For the wound inoculation assays, 15 leaves per genotype from three-week-old rice seedlings were gently pricked with a sterile needle to generate three wound sites per leaf, and the wound sites were inoculated with 10 µL drops of the conidial suspension, after which the inoculation sites were covered with moist cotton soaked in a preservative solution. For the spray inoculation assays, two-week-old seedlings were uniformly misted with a conidial suspension (2.0 × 10^5^ spores/mL) prepared by eluting spores from culture plates with 5 g/L gelatin. In both assays, the plants were incubated in a moist chamber at 28°C in the dark for 24 h and then subjected to a 12 h light/12 h dark cycle at the same temperature and humidity for six days.

### 4.5 RNA extraction and qRT–PCR

Total RNA was extracted from two-week-old Nipponbare seedlings spray-inoculated with *M. oryzae* strain 70-15 (treatment) or 5 g/L gelatin (mock control) after 24 h of incubation at 28°C in the dark (≥ 90% humidity) by grinding liquid nitrogen-frozen leaf tissue in TRIzol reagent (Invitrogen, Carlsbad, CA, USA) and following the manufacturer’s protocol; RNA integrity was confirmed by 1% agarose gel electrophoresis, and the purity (A_260_/A_280_) was determined on a NanoDrop 2000 (Thermo Fisher Scientific, Waltham, MA, USA). For resistance gene expression, *osaca5* seedlings were processed similarly after 2 h of inoculation, and 1 µg of total RNA was reverse transcribed and preamplified in a single tube using HiScript IV All-in-One Ultra RT SuperMix (Vazyme, Nanjing, China). qRT–PCR was performed on a LightCycler 480 II (Roche, Basel, Switzerland) with ChamQ Universal SYBR qPCR Master Mix (Vazyme) in 20 µL reactions (1 µL of cDNA, 200 nM primers) using the following conditions: 95°C for 30 s and 40 cycles of 95°C for 10 s/60°C for 30 s, followed by melt-curve analysis; each of three biological replicates was assayed in technical triplicates.

### 4.6 Measurements of net Ca^2+^ flux

Ca^2+^ movement in and out of rice mesophyll cells, known as Ca^2+^ flux, was measured in real time using noninvasive microtest technology equipment (NMT Physiolyzer, YoungerUSA LLC; Xuyue (Beijing) Sci. & Tech. Co., Ltd.). The leaf samples were torn, and the resulting pieces were fixed to the bottom of a culture dish with double-sided tape. Measuring solution was added to submerge the samples, and after a 30-minute incubation, the solution was discarded and replaced with 5 ml of fresh measuring solution for analysis. A mesophyll cell was identified under a microscope, and the Ca^2+^ flux microsensor was positioned approximately 10 μm from the cell surface before detection. Data were collected on each mesophyll cell for 5 minutes, and a treatment solution was added to the culture dish to reach a final concentration of 1 μM. Ca^2+^ flux was continuously monitored until the signal stabilized and did not significantly increase or decrease. Each test group consisted of 6 replicates. Ca^2+^ flux data were recorded using imFluxes V2.0 software (YoungerUSA LLC, Amherst, MA 01002, USA), and the expressed as picomoles • cm^−2^ • s^−1^; a positive value indicates efflux, and a negative value indicates influx.

### 4.7 DAB and NBT staining

To detect the accumulation of reactive oxygen species in rice following inoculation with *M. oryzae*, histochemical staining using DAB and NBT was performed to assess the localization of hydrogen peroxide (H_2_O_2_) and superoxide anion (O_2_^−^), respectively, and quantify their concentrations. Rice leaves from two-week-old seedlings were harvested at 24 hours (for NBT) or 48 hours (for DAB) postinoculation with *M. oryzae* strain 70-15 (at a spore concentration of 1.5 × 10^5^/mL) and immersed in the respective staining solutions (DAB: 1 mg/mL, pH 3.5; NBT: 0.1%, pH 7.8). The samples were vacuum-infiltrated at –0.08 MPa to –0.1 MPa for 30 minutes and subsequently incubated in the dark at 25 °C for 8 hours (DAB) or 12 hours (NBT), after which the DAB-treated samples were gently agitated at 75 rpm. Chlorophyll was removed by boiling the leaves in 95% ethanol for 20 minutes. DAB-stained samples were imaged using a Smartzoom 5 stereomicroscope (Carl Zeiss AG, Oberkochen, Germany), where reddish-brown precipitates indicated sites of H_2_O_2_ accumulation, and the pixel intensity of the stained areas was quantified using ImageJ. NBT-stained samples were photographed with a smartphone, with blue deposits revealing O_2_^−^ production, and the percentage of stained area per leaf was calculated in ImageJ using the formula: stained area (%) = (blue pixels area/total leaf pixels) × 100%.

### 4.8 Detection of ROS levels

ROS were detected using the luminol method. Rice plants grown on 1/2 MS medium for 10 days were selected, and sheaths approximately 3 mm in length were excised and equilibrated in ddH_2_O for 12 hours. The sheaths were then treated with 1 µM flg22 in a solution containing 20 µM luminol (Sigma) and 10 µg/ml horseradish peroxidase (Sigma). The reaction was continuously monitored at 5-second intervals for 15 minutes using a microplate reader (Tecan, Switzerland).

### 4.9 Statistical analysis

Lesion area percentages and lesion lengths were measured on digital images using ImageJ software. Agronomic traits such as plant height and tiller number were recorded and compiled for statistical evaluation. Sample normality for agronomic trait measurements and lesion data was assessed by the Shapiro–Wilk test in GraphPad Prism 8.0. Quantitative datasets were subjected to one-way analysis of variance (ANOVA) to detect overall differences among treatment groups, followed by Dunnett’s multiple comparisons test to compare each treatment with the control and report multiplicity-adjusted p values. All the statistical analyses were performed using GraphPad Prism. Differences at *p* < 0.05 and *p* < 0.01 were considered significant and highly significant, respectively.

## Data Availability

The raw data supporting the conclusions of this article will be made available by the authors, without undue reservation.
