# Supplementary material for: The endoplasmic reticulum-localized Ca2+-ATPase OsACA5 regulates immunity and the seed setting rate in rice
Source: Front Plant Sci. 2026 Feb 5;17:1758629. doi: 10.3389/fpls.2026.1758629 (PMC12916595; doi:10.3389/fpls.2026.1758629)
Supplement: Supplementary file 1 [file DataSheet1.docx]

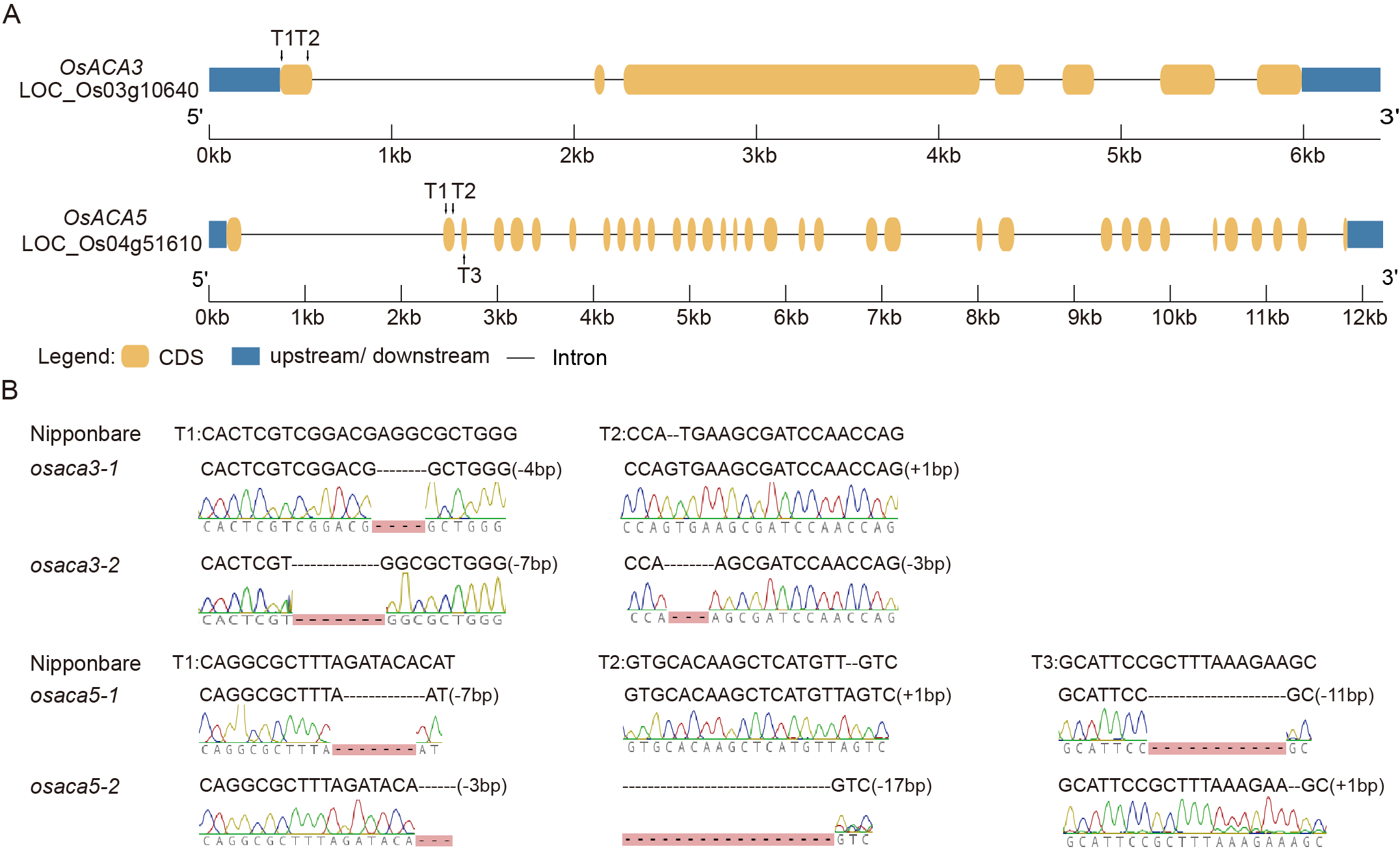


**Figure S1**. Generation and identification of *osaca5* and *osaca3* mutant lines. (A) Schematic representation of the *OsACA5* and *OsACA3* gene structures showing untranslated regions (UTRs), exons, and introns. The positions of the CRISPR/Cas9 target sites (T1-T3) are indicated. (B) Comparison of the mutant alleles with the corresponding wild-type (WT) alleles. With respect to *OsACA5*, deletion mutations in the *osaca5-1* and *osaca5-2* alleles are shown. With respect to *OsACA3*, deletion mutations in the *osaca3-1* and *osaca3-2* alleles are shown. Deleted nucleotide regions are indicated by dashed lines.


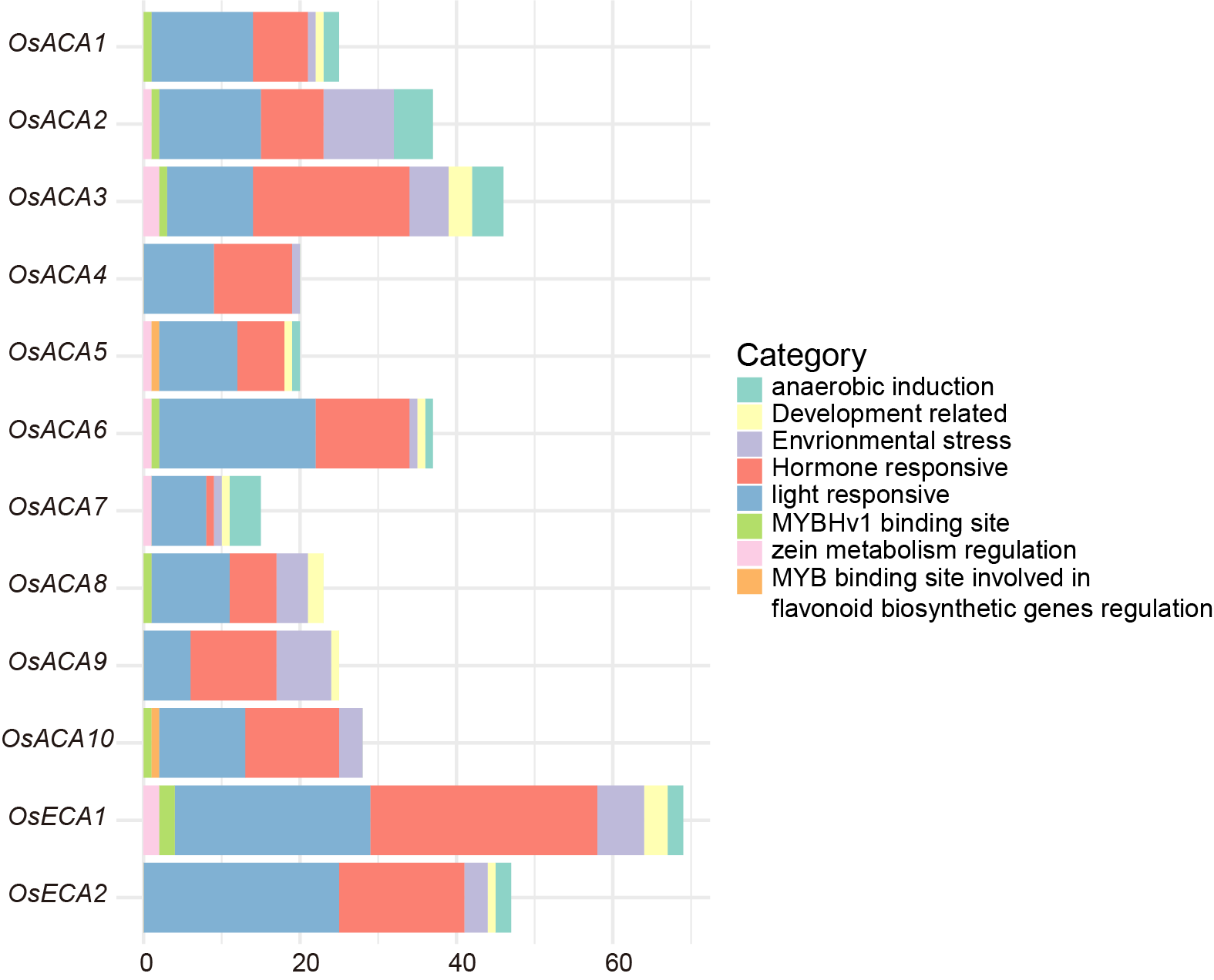


**Figure S2.** Analysis of *cis*-acting elements in the promoters of P-type Ca²⁺-ATPase genes. The 2.0 kb genomic sequences upstream of the transcription start sites of the *Os*ACA genes were analyzed using the PlantCARE database. The identified *cis*-acting elements are categorized by function and color coded accordingly. The type, number, and relative position of these regulatory elements within each promoter are displayed. The x-axis represents the total number of different *cis*-acting regulatory elements present in the promoter regions of the indicated genes.


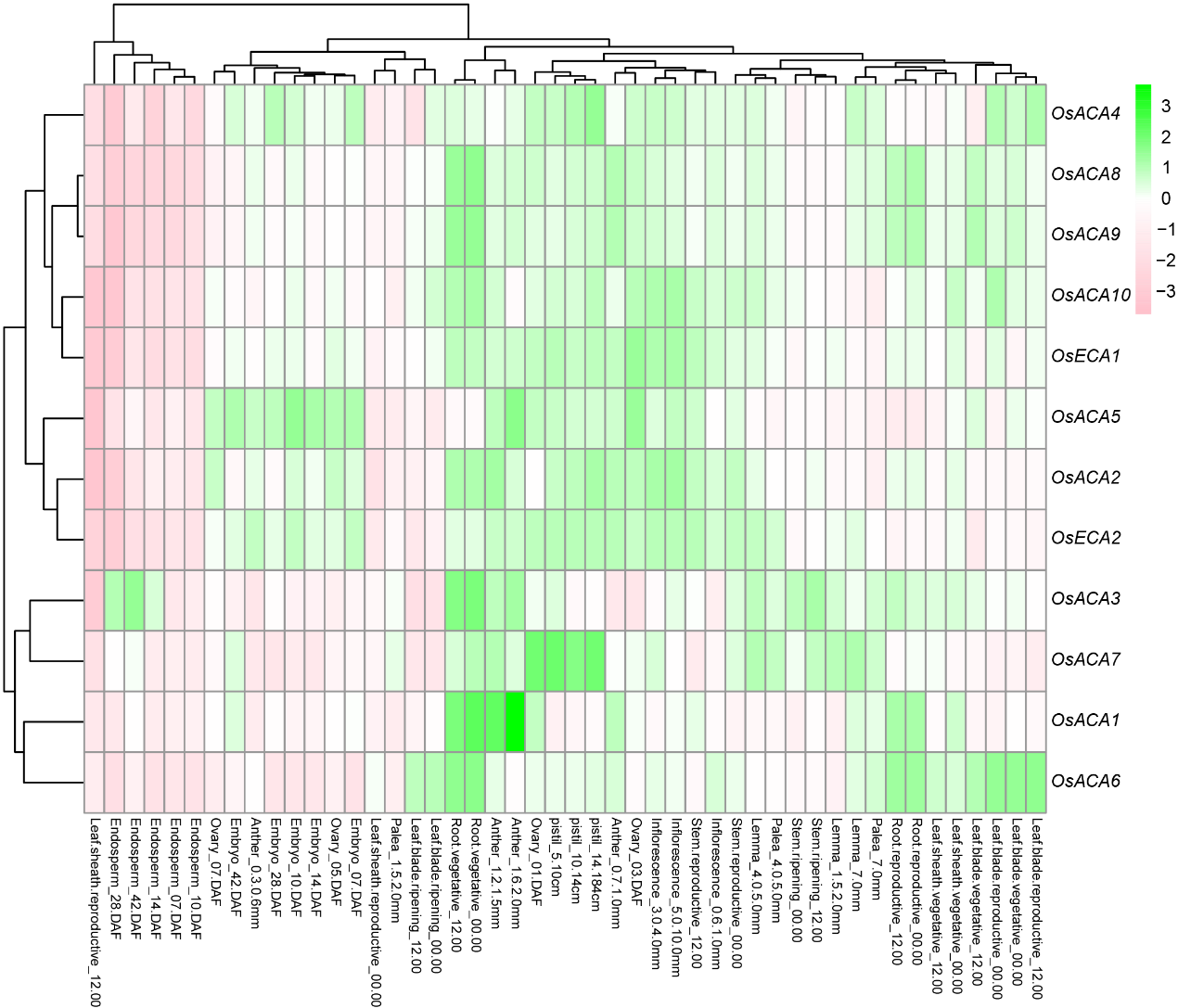


**Figure S3**. Spatiotemporal expression profiling of P-type Ca²⁺-ATPase genes. The expression patterns of the *Os*ACA genes across various tissues and at different developmental stages were obtained from the RiceXPro database. The heatmap displays the log2-transformed transcripts per million (TPM) values, with red and blue indicating high and low expression, respectively.


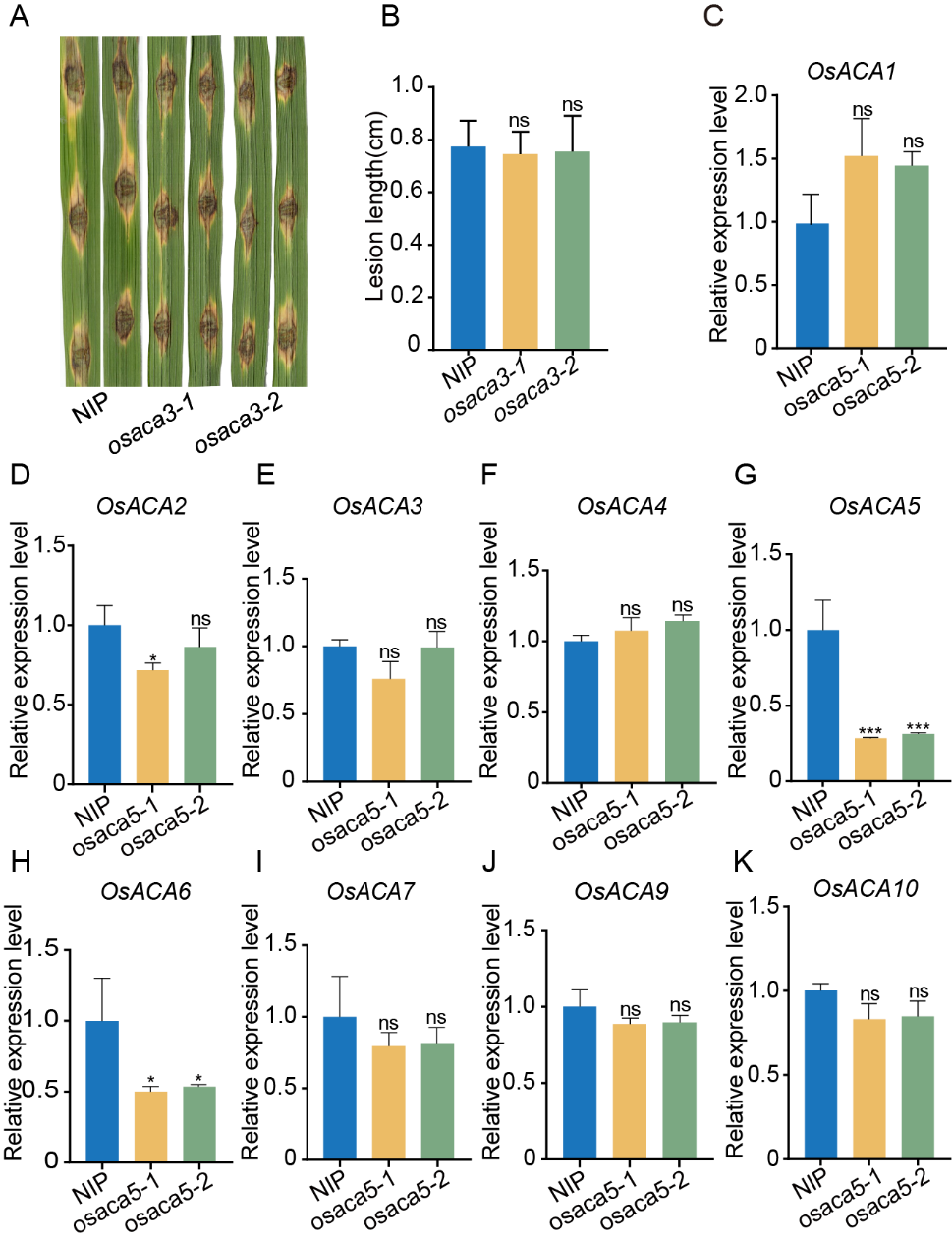


**Figure S4.** Wound inoculation phenotypes of *osaca3* mutants in response to *Magnaporthe oryzae* infection.(A) Representative lesion symptoms on the leaves of Nipponbare (NIP) and two independent *osaca3* mutant lines (*osaca3-1* and *osaca3-2*) following wound inoculation with *M. oryzae* strain 70-15. (B) Quantification of lesion length (cm) for NIP and *osaca3* mutants. The data are presented as the mean ± SD. Statistical significance was determined by one-way ANOVA; ns indicates no significant difference compared with NIP. (C-K) Expression analysis of OsACA family genes (*OsACA1*-*OsACA10*) in the *osaca5* mutant background. *OsACA8* and *OsACA9* were not included because of their high sequence similarity.

**
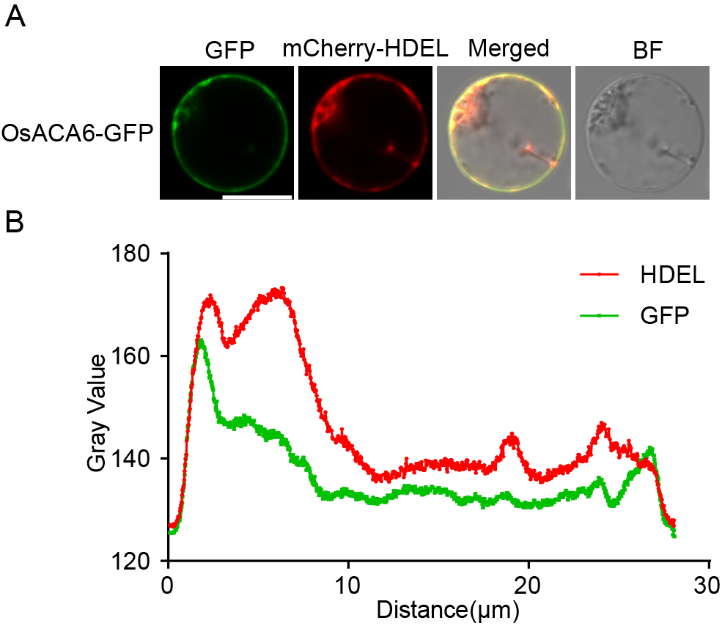
**

**Figure S5** Subcellular localization of *OsACA6* and coexpression with the ER marker HDEL. (A) Confocal images of OsACA6-GFP (green) and HDEL (red). Merged, merged channels; BF, bright field. Scale bar = 10 μm.(B) Line-scan fluorescence intensity profiles along the indicated line show nonmatching peaks of GFP and HDEL signals, suggesting no colocalization. Fluorescence intensity was measured across the entire protoplast.


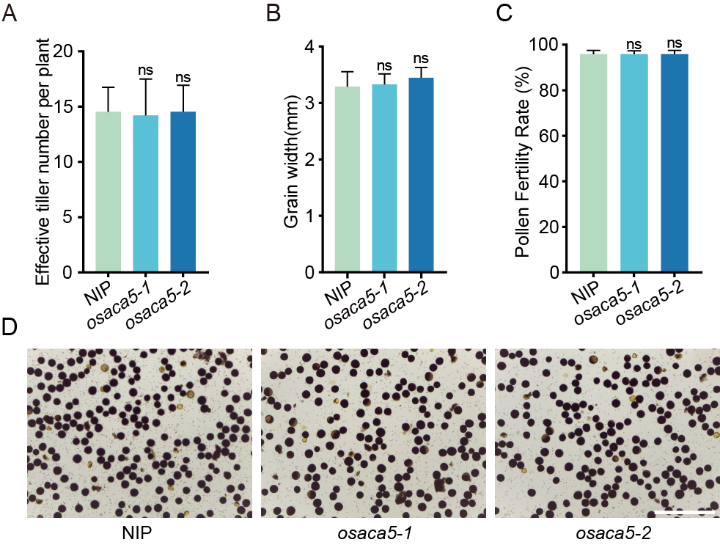


**Figure S6** Analysis of the agronomic traits of and pollen viability in *osaca5* plants. (A) Effective tiller number of NIP plants and *osaca5* mutants. (B) Grain width comparison. (C) Statistical analysis of pollen viability determined by iodine staining. (D) Representative images of pollen grains stained with iodine solution; scale bar = 200 μm. The data in A-C are presented as the mean ± SD (n ≥ 15). Not significant, *p*>0.05.
