# Supplementary material for: The endoplasmic reticulum-localized Ca2+-ATPase OsACA5 regulates immunity and the seed setting rate in rice
Source: Front Plant Sci. 2026 Feb 5;17:1758629. doi: 10.3389/fpls.2026.1758629 (PMC12916595; doi:10.3389/fpls.2026.1758629)
Supplement: Supplementary file 2 [file Table1.docx]

**Table 1** Primers used for genotyping and vector construction in this study.

| Primer Name | Primers(5‘-3’) |
| --- | --- |
| For CRISPR/Cas9 | |
| OsACA5--Cas9-F | GTTTCTTCAATCAAGTTTTACGC |
| OsACA5-Cas9-R | GTTTAACAAGTTACACGAAACTC |
| Vector Construction | |
| OsACA5-1390-GFP-F | TCTGCACTAGGTACCTGCAGATGGAGTCCGCGTCGTCT |
| OsACA5-1390-GFP-R | ATGGATCCGTCGACCTGCAGTACATCGCTGTGATCAGC |
| OsACA3-1390-GFP-F | TCTGCACTAGGTACCTGCAGATGGAGAGCTACCTGAAG |
| OsACA3-1390-GFP-R | ATGGATCCGTCGACCTGCAGGAAATCAACTGGGATCAG |
| OsACA10-1390-GFP-F | TCTGCACTAGGTACCTGCAGATGGAGAGCTACCTG |
| OsACA10-1390-GFP-R | ATGGATCCGTCGACCTGCAGCGACGAGCCGACGGGAA |
| OsACA6-1390-GFP-F | TTCTGCACTAGGTACCTGCAGATGGAGGGCGGGAGAAGC |
| OsACA6-1390-GFP-R | CATGGATCCGTCGACCTGCAGTAGAATCTCTGGGCCTTCAGGA |

**Table 2** Primers used for quantitative RT–PCR (qPCR) analysis in this study.

| Primer Name | Primers(5‘-3’) |
| --- | --- |
| OsACA1-RT-F | CATCTCTGCGTCCTTCAC |
| OsACA1-RT-R | TCATCATCGCGTCGTTCG |
| OsACA2-RT-F | GCTTACAGCTATGACAAGGG |
| OsACA2-RT-R | ACCTCATCTCCATGGACTCC |
| OsACA3-RT-F | TGAAGGTGGCATTGCCATT |
| OsACA3-RT-R | TGGTGAAGATCTAGCCATCA |
| OsACA4-RT-F | GGGTGCTGAGTTTAGAGAGA |
| OsACA4-RT-R | GCAAGTGCTTCACAAGTGTA |
| OsACA5-RT-F | TGGTGATGTTGTGCCACTTA |
| OsACA5-RT-R | TCCGGTCATGCTGGATTCA |
| OsACA6-RT-F | CGGAGGATGGAATAGCAATA |
| OsACA6-RT-R | TCCATAGGCAAGGAGCGA |
| OsACA7-RT-F | GCGTTCGCCTACAAGCAAGT |
| OsACA7-RT-R | TGGTCTGCACGGGTCTTTC |
| OsACA9-RT-R | CGGGACGACGACGATGGC |
| OsACA9-RT-R | CGCGCTTGGCGGGGATGT |
| OsECA1-RT-F | ACGATCTCTTGTTCAGAAACCT |
| OsECA1-RT-R | CAAGTTCTTATTCCACAAGCCC |
| OsECA2-RT-F | TGCAGGAAAAGATACCCTAAGG |
| OsECA2-RT-R | TGCCTCATCATCATAGCTAAGG |
| OsCNGC1-RT-F | GTTCGTCATTCTTCCCGTAATG |
| OsCNGC1-RT-R | ACTCGAACAGGAACGACAC |
| OsCNGC2-RT-F | TATTTGCTGTTTTCGTCTCGAC |
| OsCNGC2-RT-R | ATTGTTCATGACGCAGTATTCG |
| OsCNGC3-RT-F | GAAATAACATTCGCAACCGTCA |
| OsCNGC3-RT-R | CCATCGTCGCTTGCATATTG |
| OsCNGC4-RT-F | TCACATTTTTATGCGCTAGAGC |
| OsCNGC4-RT-R | TGTAGTTCCTCCAGTTTGGTAC |
| OsCNGC5-RT-F | GCAGTCTGTATTTTTGGGAGTC |
| OsCNGC5-RT-R | GCACAACGTATGCACAGTATAG |
| OsCNGC6-RT-F | GAGAAGCTGTGGGTGATTAATG |
| OsCNGC6-RT-R | ATACCTCGTAGCCGCCATTAAT |
| OsCNGC7-RT-F | CATATGCAATGCAACCGTACG |
| OsCNGC7-RT-R | GCAGGATACGATCGTACTACAA |
| OsCNGC8-RT-F | TCGATGAAGCTGACTAACAAGA |
| OsCNGC8-RT-R | TTGCGTGGTTACAAACTTACAG |
| OsCNGC9-RT-F | AATTGCTGTTGTACAACGAAGC |
| OsCNGC9-RT-R | TGGAGAGCTCGCCTGACAT |
| OsCNGC10-RT-F | ATTTGCTACTGTGAAATCACCG |
| OsCNGC10-RT-R | ATCAGTTACCTGTCAACCCAAA |
| OsCNGC11-RT-F | CTTGGAGCAATCTTCAGTGAAC |
| OsCNGC11-RT-R | TTGTACCTAGCACAAGTGGATT |
| OsCNGC12-RT-F | CTTGGAGCAATCTTCAGTGAAC |
| OsCNGC12-RT-R | TTGTACCTAGCACAAGTGGATT |
| OsCNGC13-RT-F | GCGTCTTCTCCTACAACTTTTG |
| OsCNGC13-RT-R | CAACCAAAATAACCACCTGAGG |
| OsCNGC14-RT-F | CAGATCAATGGATGTCATATCG |
| OsCNGC14-RT-R | GGAGGTTCATAAGCAAGAGT |
| OsCNGC15-RT-F | ACCAGTTCAGAAGACTACACAG |
| OsCNGC15-RT-R | CACCATGAGTATGATTCCCACC |
| OsCNGC16-RT-F | GAGGAGCTCTACTACATGCAG |
| OsCNGC16-RT-R | CGCTCTTCTTGGTGTTCTTG |
| OsCNGC17-RT-F | GAGTACTGATTGGTAGTGTCGA |
| OsCNGC17-RT-R | GCCAGACTGACAAAAGTATAATTGG |
| OsCAX1-RT-F | CTTGACTTCAAGCTGCTAGAGA |
| OsCAX1-RT-R | GTAATGTGATGTTCCATCCTGC |
| OsCAX2-RT-F | CGTTGTTCAACAATGTCTGCTA |
| OsCAX2-RT-R | CCGAATTGAGCTTCTGAATGAG |
| OsCAX3-RT-F | CGGATGATGATGGAGTCGAATA |
| OsCAX3-RT-R | GAGGAAGATGAGAGGTTGTGTG |
| OsCAX4-RT-F | GTGTTTGCCTTCAGCTTGATAG |
| OsCAX4-RT-R | ACAGTATTGGCAATATGTTCGC |
| OsCAX5-RT-F | TCGCTACTAGGCTCTATACTGT |
| OsCAX5-RT-R | AAGCAATCCTGAGTTTACAACG |
| OsCAX6-RT-F | GAATAACACCTTTAGCTGAGCG |
| OsCAX6-RT-R | GCATTTCCGAACGTAGCATTTA |
| OsCAX7-RT-F | GTAGCAGAGACGAAGTTCTACG |
| OsCAX7-RT-R | CGAATTGCAAGTTTCCTGACAT |
| OsCAX8-RT-F | CTACCTTAGCGCCGAGATATAC |
| OsCAX8-RT-R | GTATACCACTAAGCCCACGAAG |
| OsCCX1-RT-F | TCCACGGAGATTTTTATTCCCA |
| OsCCX1-RT-R | ATCGAATTACCCCATGCTAACA |
| OsCCX2-RT-F | ACCGCGTCCGAGTACTTCT |
| OsCCX2-RT-R | CAACAACGGTGGACACGAA |
| OsCCX3-RT-F | CTTGTGCTTTCTTTGCATCAAC |
| OsCCX3-RT-R | CGGGCCCCTTATGAAAAGAATA |
| OsCCX4-RT-F | ATCATCACTAAGCTCCAACCTC |
| OsCCX4-RT-R | TGAAGAAGATGTCAGCCTCATT |
| OsEFCAX1-RT-F | GATCATGGATGACTTTGACACG |
| OsERCAX1-RT-R | CTTGTTCTCGCATGGTAATCAG |
| OsEFCAX2-RT-F | GGAAGAAGAAACGGACTTTGTC |
| OsERCAX2-RT-R | GATGATGAGTACCTCCGATGAG |
| OsMHX1-RT-F | GCAGCAATTCGGTGAACATATA |
| OsMHX1-RT-R | TTTCTAGACCATGAGAGCGAAC |
| OsMHX2-RT-F | GTGCGTTTATTTATACCGCTGT |
| OsMHX2-RT-R | GCTCTCCATCGATTTGAAGAAC |
| OsNCKX1-RT-F | GGTCGGAGAAGGACTTGT |
| OsNCKX1-RT-R | ACTCCTCCTCCTCCTCTAC |
| OsANN1-RT-F | AAGTACTTCGTGAAGGTGCTAA |
| OsANN1-RT-R | GGAAACTCTTGTAGTCACCAGA |
| OsANN2-RT-F | GATCCACGGCAAATTCGAGAG |
| OsANN2-RT-R | GCCCATCATAGCGATATACAGT |
| OsANN3-RT-F | TCAAGCACTACAAGGAAATCCA |
| OsANN3-RT-R | AACACAACAACAACAACAGCAG |
| OsANN4-RT-F | GTGATCCGGACATCGATTCTAG |
| OsANN4-RT-R | TGTTGTACCTGACCTTGTACTC |
| OsANN5-RT-F | GTACCAGAAGAGGAACAGTGTC |
| OsANN5-RT-R | CCGTACACTGGAAAAACAACAT |
| OsANN6-RT-F | TCGATCGATCATGTCCATCAAT |
| OsANN6-RT-R | CTTTCCAGAAATCGAGGGAGAG |
| OsANN7-RT-F | TGGACATGCAGTACATCAAGG |
| OsANN7-RT-R | CTTAACACACGCTCAAAGAGAC |
| OsANN8-RT-F | TACTGTCGCAGCAGAAGA |
| OsANN8-RT-R | TCGAACCAGACAAGATTGAA |
| OsANN9-RT-F | CGAGACCTCAGGAAATTATCGA |
| OsANN9-RT-R | TACAGTTTCCAGGTAGAACCAC |
| OsANN10-RT-F | ATCGGAGAGGCAGAAAATCAAG |
| OsANN10-RT-R | GGTTGACCATGAGGATTTTCTG |
| OsGLR1.1-RT-F | GCCATTAACAGCTGACTTATGG |
| OsGLR1.1-RT-R | GCTCAAGCTTGCAGTATAACTC |
| OsGLR1.2-RT-F | GACCAGAAGGACCTCAAGTC |
| OsGLR1.2-RT-R | TCGTGGAGATGCTGAACG |
| OsGLR1.3-RT-F | AAGAAGTTCCTCGAGGCGATC |
| OsGLR1.3-RT-R | GATGTTCAGCACGCGGAAG |
| OsGLR2.1-RT-F | GGTAGAGGCATCATACCATACC |
| OsGLR2.1-RT-R | AACTTGTAGAGTTCTCGCTCAA |
| OsGLR2.2-RT-F | GCCAGTCTTCCATATTTCGTTC |
| OsGLR2.2-RT-R | GTGCACAATAAAGACCCTTGTT |
| OsGLR2.3-RT-F | CAACCTCAGCCCTTTTAATAGC |
| OsGLR2.3-RT-R | CTGGTTGGAGTCTATTGTCCTT |
| OsGLR2.4-RT-F | GGGATTCCAATGGAGACGATAT |
| OsGLR2.4-RT-R | TGTTGCAGTGAAGGAGATAACT |
| OsGLR3.1-RT-F | GGTGTTTCGATGATTGCTATCC |
| OsGLR3.1-RT-R | TATGTGCAATTGTTGAGCACTG |
| OsGLR3.2-RT-F | TGGTGTTCTCGCTTTCTAATCT |
| OsGLR3.2-RT-R | TTCATCCTTCAACGTAACAACG |
| OsGLR3.3-RT-F | GGCGTGAGGAATCATATCT |
| OsGLR3.3-RT-R | TCTCGTCTTGTCGTTGTC |
| OsGLR3.4-RT-F | GAGCTCTTCCTCTCCATCAAC |
| OsGLR3.4-RT-R | CAATTCATCAGATCGGATTGCC |
| OsGLR3.5-RT-F | GTAGGTGTACTTGTCAATTCGC |
| OsGLR3.5-RT-R | CATCATCATCACCATCGGTTTC |
| OsGLR3.6-RT-F | CTTCTTCCTTTTCCTCCGTCTTA |
| OsGLR3.6-RT-R | ACACGGCTAATCATCGGATAAT |
| OsGLR3.7-RT-F | TATAAACTCAAGCTACACGGCA |
| OsGLR3.7-RT-R | TTCTCGCAAAAGATCCAACTTG |
| OsGLR3.8-RT-F | GCTCTTCCTCTCCATCAACT |
| OsGLR3.8-RT-R | TCTCTAGCCTCCTCCTCCT |
| OsGLR4.1-RT-F | GCATGAAAAGAGGAGAAGTTGG |
| OsGLR4.1-RT-R | TAGTCGCCCTTCTTTAATTCGT |
| OsGLR4.2-RT-F | AGTGGGAGTGAGAAATCTAACG |
| OsGLR4.2-RT-R | ACATCACCCACTGTAGCATTAA |
| OsGLR4.3-RT-F | GAAGACCTTATCAAGAACGCAC |
| OsGLR4.3-RT-R | GGGGTGGATGATGTGATGAC |
| OsGLR4.4-RT-F | CACAAACTGGTCAGAAAGTCAG |
| OsGLR4.4-RT-R | TCAGAATTCAGCAGTCTGTCTT |
| OsGLR4.5-RT-F | CCATCCAGGTTAAGGTCTTAT |
| OsGLR4.5-RT-R | CCGACACTCCTCCATTCA |
| OsGLR4.6-RT-F | TTCCTATTCACTGCCTTGG |
| OsGLR4.6-RT-R | GGCTGGAACCTCTTAACA |
| OsGLR4.7-RT-F | TGAGCGTGCTCTGGAAATATAA |
| OsGLR4.7-RT-R | ACATCCCAGACTAATTTCTGCA |
| OsGLR4.8-RT-F | CATCACTGGATGTATCTCAGCT |
| OsGLR4.8-RT-R | GTTTTGTGCTGAGTTACGTTCT |
| OsGLR4.9-RT-F | CTTGCTTAACTGTGTAAGCCTC |
| OsGLR4.9-RT-R | GTTCGTCGTGTTTGTGCTATAT |
| OsTPC1-RT-F | TGTTTTGGCCCTTTCACTATTG |
| OsTPC1-RT-R | TAACGTGGTACCATATGACGAG |
| OsActin-RT-F | AACCAGCTGAGGCCCAAGA |
| OsActin-RT-R | ACGATTGATTTAACCAGTCCATGA |
| OsNAC4-RT-F | TCCTGCCACCATTCTGAGATG |
| OsNAC4-RT-R | TTGCAGAATCATGCTTGCCAG |
| OsWRKY45-RT-F | AATTCGGTGGTCGTCAAGA |
| OsWRKY45-RT-F | AGTAGGCCTTTGGGTGCTT |
| OsPR3-RT-F | CTTGGACTGCTACAACCAGA |
| OsPR3-RT-R | CATTGTGGGCATTACTGATG |
| OsPR1a-RT-F | CAAAGGTCATTGTGCTCTTTGA |
| OsPR1a-RT-R | CTGAAGTCGTCGATGATCTGAG |
| OsPAL1-RT-F | CTACCCGCTGATGAAGAAGC |
| OsPAL1-RT-F | GAACCTTGTTCAGCTCCTCG |
| OsCPK12-RT-F | ATCCTTCTATGCGGGACG |
| OsCPK12-RT-R | ACTTGGTTGACCAGTATAGC |
| OsCML16-RT-F | GAGATCGAGAGGGTCTTCA |
| OsCML16-RT-R | GGTGTCGAGCTCGTTCAT |
| OsCIPK31-RT-F | GGGTGTGTACCACAGAGA |
| OsCIPK31-RT-R | CCTCAATCACCTCTGGAG |
| OsCBL2-RT-F | GAGGAGGCAGACACAAAG |
| OsCBL2-RT-R | GTCATCGACCTGGGAATG |
| OsPLC1-RT-F | TCATCACGCTCGAAGACC |
| OsPLC1-RT-R | GGCCCTTGAGGTCTTGAG |
| OsRLCK185-RT-F | GGATACTGTGCTGATGGG |
| OsRLCK185-RT-R | GCACCAGCAGCAATCTTC |
| OsCNGC9-RT-F | ACTCATGGTGGCCAGAAGAA |
| OsCNGC9-RT-R | TGCATTTGAGCAATGGCACT |
| OsCPK20-RT-F | ACGACAATGCCGTCCACC |
| OsCPK20-RT-R  OsCPK9-RT-F  OsCPK9-RT-R  OsCPK21-RT-F  OsCPK21-RT-R  OsCDPK23-RT-F  OsCDPK23-RT-R | GGTGCATCACTCCATGCT  CAAGCCGGAGAACTTCCT  GATGTAGAGGATGACGCC  CTGTTCGACCGCATCGTG  AGTCGATGACCTTGAGCG  TACTGGACGCAGACGATG  GAATCACTCCAGCAGTCC |
